# Supporting the couple relationship following dementia diagnosis: A scoping review

**DOI:** 10.1111/hsc.14006

**Published:** 2022-09-20

**Authors:** Sarah Colloby, Samantha Whiting, Alison Warren

**Affiliations:** ^1^ Livewell Southwest West Therapy Team, Cumberland Centre Plymouth UK; ^2^ Faculty of Health, School of Health Professions University of Plymouth, Peninsula Allied Health Centre Plymouth UK

**Keywords:** caregiver, dementia, relationship, resilience, wellbeing

## Abstract

There is now a significant body of research demonstrating the importance of supporting the couple's relationship for people living with dementia. Maintaining a strong relationship has been demonstrated to slow cognitive decline in dementia, reduce the caregiver's sense of burden and may delay the need for transfer into residential care. However, the potential for healthcare practitioners to deliver interventions to support the couple's relationship in the community remains largely unexplored. This scoping review aimed to locate interventions that support couples to maintain their relationship satisfaction when living with dementia. This review mapped studies across a broad range of disciplines and research methods, following the Joanna Brigg's Institute (JBI) framework. Following screening, 44 studies were identified. The approach of these programmes can be broadly grouped into three categories; Adaptation and use of shared activities to enhance the couple's relationship; Developing caregiver skills and reducing perceived burden to improve interaction and relationship quality; Connecting and strengthening the couple's relationship through sharing feelings and memories. Further research is required to explore the possibility and appropriateness of adaptation of these interventions for use by community healthcare practitioners. There is a need to identify interventions that can meet the needs of couples as dementia progresses into the moderate–severe stages. Heterogeneity and inconsistency in outcomes measurement for the couple's relationship, suggests the need to consider further how outcomes for couple's relationship quality may best be captured. It is also suggested that other existing programmes, outside of the scoping review results, but aimed at reducing dementia caregiver burden may have currently unexplored and developed outcomes for couple's relationship quality.

**What is known about this topic**

- Caregivers often experience a loss of relationship satisfaction and reciprocity as dementia progresses, which increases their sense of perceived burden.
- Maintaining the couple' relationship when living with dementia may improve the wellbeing of both the person with dementia and their partner, delay cognitive decline and the need for institutional care.

**What this paper adds**

- Highlights a range of interventions with potential for adaptation to supporting couple's living with dementia.
- Identifies the lack of interventions designed to support the couple's relationship as dementia progresses into the moderate–severe stages.
- Outlines the need for effective outcome measurement of the impact on the couple's relationship of existing interventions in dementia care.

## 1 INTRODUCTION

Over 46 million adults worldwide are estimated to have a dementia diagnosis (Alzheimer's Disease International, 2015). This incurable, degenerative brain disease, reduces a person's memory, cognitive, communicative and decision‐making abilities impacting on their day‐to‐day functioning and relationships (Alzheimer's Disease International, 2014). Informal caregiving by family members at home enables the person with dementia to remain in their home environment for as long as possible, delaying the need for transfer into residential or nursing care (Fauth et al., 2012). This has been demonstrated to offer a better quality of life for the person with dementia (Youell et al., 2016) and their spouse (Conway et al., 2018). Globally, spouses account for an average of 40% of informal carers of persons with dementia (Alzheimer's Disease International, 2018).

For many years, dementia caregiving research was based upon a deficit approach to understanding the caregiving journey (Donovan & Corcoran, 2010). This approach focused primarily on the caregivers' perceived sense of burden and loss and the need to support caregivers to develop coping behaviours (Donovan & Corcoran, 2010; McGovern, 2011). However, this model largely overlooked the positive feelings some caregivers also experience as a result of caring for their loved one (Donovan & Corcoran, 2010; Helström et al., 2007). McGovern (2011) argues that a strengths‐based approach to supporting persons with dementia and their partners, involves going beyond the concept of caregiver burden and supporting couples to maintain their sense of togetherness and maximise potential positive outcomes from the caring relationship for as long as possible.

Whilst preserving the sense of togetherness in the relationship can be challenging as dementia progresses, the couple's relationship and their commitment to it, has been identified as a resource that helps couples to make sense of their experience, find strength and weather difficulties (Merrick et al., 2013; Swall et al., 2020). Sustaining the couple's relationship and the quality of their shared life together has been found to be of primary importance to couples experiencing dementia (Helström et al., 2007). This relationship is key to sustaining a sense of identity for both caregiver and recipient (Helström et al., 2007). A strong spousal relationship can also slow cognitive decline for persons with dementia (Norton et al., 2009), and some research indicates, a delay in the need for transfer into residential care (Davies et al., 2010).

Supporting clients to maintain their participation in chosen relationships, roles and occupations is considered to be central to many healthcare providers' service, most notably, occupational therapy (Bennett et al., 2019). However, research on the importance of the couple's relationship in dementia care is sparse (Yong et al., 2020). Several randomised controlled trials (RCTs) have been conducted to evaluate the impact of interventions with couples in the community where one partner has dementia, however, these do not assess the impact of the programmes on the couple's relationship. For example, the Care of Persons with Dementia in their Environments (COPE) programme (Gitlin et al., 2010), Tailoring Activities for Persons with Dementia and Their Caregivers (TAP) (Gitlin et al., 2016) and the Community Occupational Therapy in Dementia (COTiD) programme (Wenborn et al., 2021).

A recent scoping review conducted by Bielsten and Helström (2017) identified couple‐centred interventions, which enabled the joint participation of both the person with dementia and their caregiver. However, whilst the study identified a range of intervention types, it excluded interventions aimed at the caregiver or receiver individually, that may still have benefits for the couple's relationship. This scoping review seeks to extend the work of Bielsten and Helström (2017), identifying both dyadic and non‐dyadic interventions that identify outcomes for the couple's relationship.

This scoping review sought to explore what interventions are available to support couples where one partner has a diagnosis of dementia, to maintain their relationship quality. Relationship quality and satisfaction has been defined by Spruytte et al. (2002), as couples having high levels of perceived closeness in the relationship, feelings of warmth, enjoyment of spending time together, sharing activities and low levels of conflict in the relationship.

## 2 MATERIALS AND METHODS

The aim of this scoping review is to identify literature across health and social care disciplines that discuss the impact of an intervention on the couple's relationship for couples living with dementia. The review was guided by the questions: (i) What interventions have been used with couples? (ii) What outcomes have been identified from the intervention?

### 2.1 Inclusion criteria

In order to draw together what is currently known about interventions and the couple's relationship, the authors chose to include both interventions directly aimed at supporting the maintenance of the couple's relationship and those which reported outcomes (positive, negative or neutral) for the couple's relationship as either a secondary or unanticipated outcome. Studies which focus primarily on interventions delivered in residential care facilities have been excluded due to the difference in context, needs and intervention options available (Førsund et al., 2016). Additionally, interventions aimed primarily at paid caregivers or non‐spousal caregivers have been excluded due to the difference in the relationship being supported. However, where samples have included a mix of different relationship types including spousal, they have been included in the review. Whilst during the research proposal stage, the authors considered the inclusion of grey literature, it was later excluded as database searches generated a large quantity of peer‐reviewed literature from a wide range of journals, incorporating a range of methodologies and specialisms. A full list of the inclusion and exclusion criteria can be found in Appendix 1.

### 2.2 Types of sources

The scoping review gathered information from a wide variety of sources including primary research, systematic reviews and meta‐analyses from across a range of disciplines (Peters et al., 2020). This scoping review considered both experimental and quasi‐experimental study designs including randomised controlled trials, non‐randomised controlled trials, before and after studies, and interrupted time‐series studies. In addition, analytical observational studies including prospective and retrospective cohort studies, case–control studies and analytical cross‐sectional studies were considered for inclusion. This review also considered descriptive observational study designs including case series, individual case reports and descriptive cross‐sectional studies for inclusion. Qualitative studies were also considered that adopted methodologies including, but not limited to, phenomenology, grounded theory, ethnography, qualitative description, action research and feminist research.

### 2.3 Search strategy

This scoping review followed the steps set out in the Joanna Briggs Institute (JBI) framework originally developed by Arksey and O'Malley (2005), and later refined by Peters et al. (2020) to ensure the research was conducted in a systematic manner.

A search strategy was developed in consultation with an information specialist. The search strategy included the following key terms: “dementia” OR “Alzheimer*” OR “lewy body” OR “korsakoffs” AND “dyad*” OR “marriage” OR “spous*” OR “couple” OR “partner” OR “family” OR “caregiver” OR “carer” AND “intimacy” OR “quality” OR “satisfaction” OR “resilience” OR “maintenance” OR “strong” OR “healthy” OR “wellbeing” OR “well‐being” OR “solidarity” OR “close*” OR “mental health” AND “counsell*” OR “support” OR “guidance” OR “education” OR “approach” OR “technique” OR “model” OR “care” OR “intervention” OR “program*” OR “therap*” OR “clinic*”. Searches were conducted in January 2021 within the combined fields ‘title’ and ‘abstract’, included studies published from January 2000 onwards, and those published in English language only. The databases searched included; AMED, CINAHL, Medline, PsycINFO, PubMed and Soc Index. The text words contained in the titles and abstracts of relevant articles, and the index terms used to describe the articles were used to develop a full search strategy. The search strategy, including all identified keywords and index terms were adapted for each included information source and database as determined by the platform. Duplicate sources and publications that did not directly relate to the research aims were eliminated. The title and abstract of each article were reviewed thoroughly to select the most relevant sources.

### 2.4 Study selection

**FIGURE 1:**
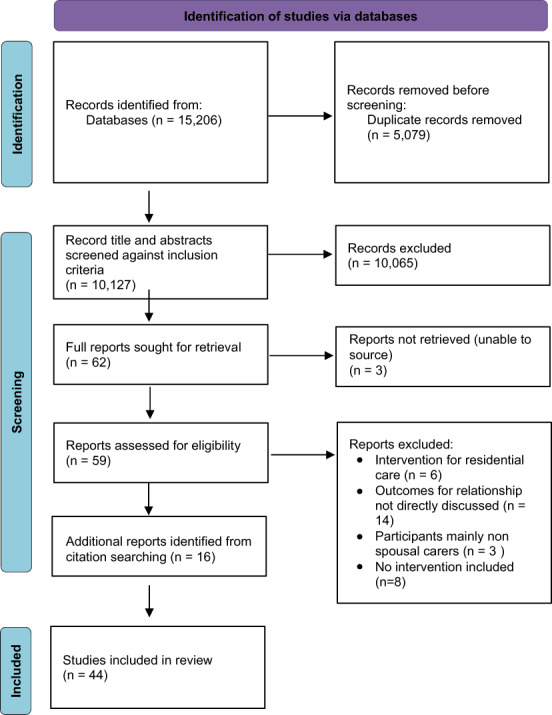
PRISMA table.

Following the search, all identified citations were collated and uploaded into the bibliographic citation management system, Endnote X8.2 (Clarivate Analytics) reference manager. In accordance with the process of the PRISMA‐ScR (see Figure 1) (Tricco et al., 2016), duplicate studies were then identified and removed and the titles and abstracts for remaining records were then screened against inclusion and exclusion criteria (see Appendix 1). The records remaining were then sought for full‐text analysis against the inclusion and exclusion criteria and charted using the charting tool. Reasons for exclusion at this stage were noted and included in the PRISMA diagram (see Figure 1). As recommended by Peters et al. (2020), following screening of retrieved articles, article reference lists were examined and further papers fitting the review's inclusion criteria were identified and added to the list of articles reviewed.

### 2.5 Data extraction

A data extraction tool pro forma was used to ensure consistency in how the data was extracted. The categories of information recorded in the tool were based upon those identified by Arksey and O'Malley (2005) in their framework for conducting scoping reviews. In addition to this, information regarding the outcomes for the couple's relationship was included to gather data pertaining to the research aims as well as an additional category of ‘intended for intervention by (Professional)’ to inform the analysis of interventions. This pro forma was piloted with three studies and reviewed by the second author to ensure it was fitting and comprehensive in gathering the data required. The contents of the completed data extraction tool pro forma were then combined into a charting table (see Appendix 2).

### 2.6 Data presentation

**FIGURE 2:**
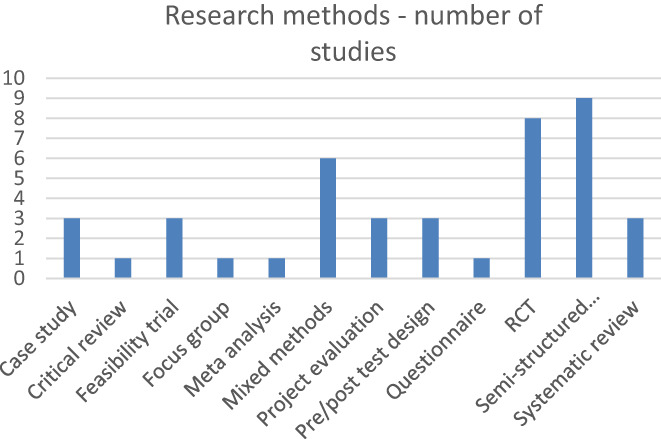
Research methods utilised by included studies.

**FIGURE 3:**
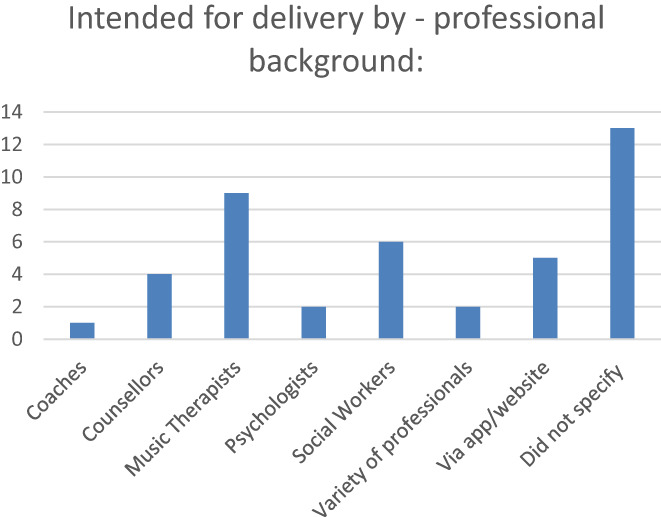
Professional background of researcher delivering intervention.

The extracted data is presented in tabular form that aligns with the aims of this scoping review. A narrative summary also accompanies the charted results and describes how the results relate to the review's aims. The findings of this scoping review are organised by intervention type and stage of dementia. This then became the overall organising structure, providing themes for the narrative summary of study findings (Peters et al., 2020). A numerical analysis of the studies included was completed to present figures and graphs displaying study types and the professions that interventions were intended to be delivered by and stage of dementia of participants (see Figures 2 and 3, Appendix 4).

## 3 RESULTS

### 3.1 Summary of papers included

The scoping review yielded 44 papers meeting the inclusion criteria, which were heterogenous in their research methods. The most frequently employed methods were qualitative studies using semi‐structured interviews (*n* = 10) and randomised controlled trials (RCTs) (*n* = 9), followed by a mixed methods approach (*n* = 6) (see Figure 2). Twelve of the studies included a standardised outcome measure for relationship quality. However, 11 different outcome measures were used across these studies (see Appendix 3), making comparison of outcomes between studies more challenging. Some of the studies included did not incorporate any assessment of relationship quality prior to the intervention, with which to compare outcomes and intervention efficacy (Dassa et al., 2020; Ingersoll‐Dayton et al., 2016). The studies cited in this scoping review were unable to assess prior relationship quality and the impact of this variable on the outcomes of, or appropriateness of, their interventions. Outcomes were typically assessed immediately after the intervention (Ingersoll‐Dayton et al., 2013), at intervals such as 2 and 4 months (Auclair et al., 2009) or up until 12 months post‐intervention (Charlesworth et al., 2016). This limited potential investigation of the sustained benefits as dementia progressed into the moderate–severe stages.

A number of studies in this scoping review identified that the outcome measures used were not very accessible for clients (Allan, 2018) or client‐centred (Wenborn et al., 2021). Moon and Adams (2012) suggest that developing client‐centred goals with caregivers and care receivers may prove more effective in planning and assessing the outcomes of interventions.

The majority of studies were conducted in the United States (*n* = 13), United Kingdom (*n* = 10) and Australia (*n* = 3). This may be representative of where studies in this field are being conducted, or may also be in part, due to the exclusion of studies not published in English.

The charting table shows that the most common intervention location was delivery within a community facility or clinic (*n* = 22), followed by interventions delivered within the participant's home (*n* = 10), web, app‐based or telephone‐based interventions (*n* = 8) and finally mixed delivery methods (*n* = 5). The majority of music‐based interventions (*n* = 10) were designed to be delivered by a trained music therapist and most counselling‐based interventions were intended to be delivered by trained counsellors, psychologists or social workers (see Figure 3). The other studies included did not specify the professional background of the facilitator. Of those interventions which detailed the stage of dementia of participants, the majority were aimed at people with either mild or mild–moderate dementia (*n* = 24) (see Appendix 4). Of all the studies included, only four studies stated the interventions were intended for delivery with persons including those with moderate to advanced or advanced dementia. Five of the studies covered interventions aimed solely at the caregiver and 11 of the studies were unclear about the stage of dementia participants had reached.

Whilst the outcomes of studies included were mixed, papers were grouped into three themes based upon the method by which they aimed to support the couple's relationship. The first theme included those interventions which aimed to enhance the couple's relationship through the use and adaptation of shared activities. The second theme covers those interventions which attempted to improve the couple's relationship, by developing caregiver skills and reducing caregiver's sense of burden. Finally, the third theme summarises interventions which aimed to strengthen the couple's relationship by enabling couples to talk about their feelings and share important memories together.

#### 1. Adaptation and use of shared activities to enhance the couple's relationship

The scoping review located several papers which discuss interventions that deliver or adapt activities to support caregivers and care receivers to engage in meaningful and enjoyable activities together. These interventions include the use of leisure and physical recreation activities, arts, music and singing to enhance the couple's relationship.

Carbonneau et al. (2011) examined the impact of a multi‐modal leisure education programme which included caregiver education and supported the caregiver to share and adapt leisure activities to enjoy with the person with dementia as a couple. Whilst there was no significant impact on caregiver wellbeing, there was a statistically significant improvement in relationship quality (Carbonneau et al., 2011). Participants reported that the programme enabled them to experience more pleasurable moments and improved their interactions, self‐efficacy and ability to enable the person with dementia's participation. The community‐based ‘Paired PLIÉ’ (Preventing Loss of Independence through Exercise) programme combines physical movement activities with mindful body awareness for both members of the dyad and provides monthly caregiver education in the home (Casey et al., 2020). Caregivers reported improvements in relationship quality and feelings of closeness (Casey et al., 2020). In contrast, a meta‐analysis of RCTs which included tailored cognitive rehabilitation and stimulation activities for persons with dementia did not identify any significant positive outcomes for caregiver‐receiver relationship quality (Leung et al., 2017).

A smartphone app named ‘DemPower’ has also been designed to support users to identify activities they can still enjoy together. The app also offers the facility to store reflections and memories and offers self‐management techniques for couples at home experiencing dementia (Bielsten et al., 2020). Couples reported that initially, using the app felt uncomfortable, but over time, it began to help them to communicate more openly and improved their relationship. It encouraged them to try new activities together and this enhanced feelings of ‘closeness’ (Bielsten et al., 2020). Another app, ‘Go & Grow’, aimed solely at the caregiver, has also been developed to provide fitness challenges for caregivers, as well as opportunities to interact with other caregivers and share experiences (Lin et al., 2020). Caregivers reported that the app offered a break from caregiving activities and assisted them in stress relief and self‐care (Lin et al., 2020). Using the app also had benefits for their relationship, enabling them to develop greater empathy and patience for their loved one, which improved the quality of interaction in the relationship (Lin et al., 2020).

The scoping review identified two studies which found benefits for the couple's relationship through engagement in arts activities exclusively. Hunt et al. (2018) found engagement in arts activities offered caregivers respite from caring responsibilities, and social connection with others in the community. Caregivers reported that this enabled them to maintain greater resilience and a positive emotional connection with their partner. Some caregivers also enjoyed making art together with their family member and found this facilitated closeness in the relationship and recovery of aspects of the pre‐dementia relationship that they had considered lost. Tyack et al. (2017) explored if arts‐based interventions for couples experiencing dementia could be facilitated using a touch screen tablet. Whilst some participants identified a positive benefit for the relationship – enabling deeper conversation and providing a focus for spending time together, others reported no change in relationship quality and some participants experienced difficulty using the technology (Tyack et al., 2017).

The scoping review also highlighted six studies utilising community‐based singing and music therapy groups, led by trained music therapists. Whilst these programmes were enjoyable for both caregiver and care receiver, clear and measurable positive outcomes for the couple's relationship were lacking in four of the studies (Allan, 2018; Clark et al., 2018, 2020; Lee et al., 2020). However, two UK‐based studies have reported positive outcomes for the couple's relationship. The ‘Carers Create’ programme by Skingley et al. (2020) enabled the continuation of singing activities by caregivers in the home environment following group participation. Caregivers reported that the group sessions offered an escape from caregiving, an opportunity to enjoy being together and enabled them to feel in touch with the person they once were, enhancing the sense of equality in the relationship. The second study assessed the impact of a group singing activity for both persons with dementia and their carers. The study reported outcomes for participants including a reduction in feelings of social isolation, meaningful interaction between caregiver and receiver and a positive impact on wellbeing for both (Osman et al., 2016). It offered an activity that the authors state supports social inclusion for all participants, regardless of the stage of their dementia (Osman et al., 2016).

The scoping review also highlighted studies delivering weekly or twice weekly music therapy by a qualified music therapist, with the couple in their own home. The intention was that through completion of the programme, caregivers would gain skills enabling them to continue to use music with their partner independently, providing ongoing benefits to the couple and their relationship (Baker et al., 2012, 2019). However, Hanser et al. (2011) identified that caregivers need further ongoing support from a Music Therapist in order to maintain this intervention with their loved ones and overcome any challenges as dementia progresses.

Home‐based music therapy sessions have successfully been combined with fortnightly counselling sessions with the caregiver in one trial (Dassa et al., 2020). Participants reported that it enabled reminiscence, laughter and enjoyment together as a couple, and a greater feeling of equality, warmth and intimacy in the relationship (Dassa et al., 2020). However, no measures were taken of relationship quality or changes to the relationship over the course of the intervention. Baker et al. (2012) trialled combining a home‐based singing and music intervention with gentle movement activities. Whilst participants reported some benefits for the couple's relationship in terms of closeness and reciprocity, a ceiling effect occurred, with outcome measures finding an existing high level of relationship quality amongst participants prior to intervention (Baker et al., 2012).

Additionally, Raglio et al. (2016) found that music and singing could be used within the home to calm participants with dementia, reducing the incidence of behavioural disturbances which then had a beneficial impact on the couple's relationship. Melhuish et al. (2019) found that music and singing also had a calming effect which assisted the completion of daily care activities, reducing caregiver strain and providing positive benefits for the couple's relationship. A further RCT is currently underway aimed at utilising the calming effects of music to benefit wellbeing and relationship quality (Baker et al., 2019).

However, a meta‐analysis of cognitive stimulation and rehabilitation RCTs by Leung et al. (2017) identified no positive impact on caregiver anxiety, sense of burden or relationship quality Leung et al. (2017) posit that this may be due to a negative effect of emotional demands in closer caregiving relationships. However, this is not supported by the critical review completed by Moon and Adams (2012) which concluded that improved communication and closeness within the caregiving relationship, facilitated by dyadic interventions increase wellbeing for both caregiver and care‐receiver.

#### 2. Developing caregiver skills and reducing perceived burden to improve couple interaction and relationship quality

Several papers focussed on the delivery of psychoeducation and skills development for caregivers, however, the outcomes have been mixed. The SAVVY Caregiver Program offers a 12‐h psychoeducational intervention, focussed on the caregiving role, skills, attitudes and self‐care (Hepburn et al., 2003). The study found that as caregivers' knowledge and confidence improved, not only did their sense of burden reduce, but their emotional response towards caring for their family member also improved and this had a positive effect on how they interacted with and responded to the care‐receivers' behaviours (Hepburn et al., 2003). Judge et al. (2009) claim to build on the successful elements of caregiver training programmes such as SAVVY and combine it with cognitive rehabilitation for persons with dementia through a skills‐based education programme called ‘ANSWERS’. In the follow‐up paper analysing outcomes for caregivers following participation in the ANSWERS programme, participants reported a reduction in caregiver strain, strain on the relationship and improved emotional health and wellbeing (Judge et al., 2013). An RCT of a telephone‐based intervention aimed at caregivers alsp reported improvements in relaionship satisfaction (Au et al., 2019).

However, other programmes have been less successful. An RCT examining the outcomes of a caregiver skills training programme aimed at improving relationship quality through increasing congruence between caregiver expectations and the functional abilities of persons with dementia did not find any lasting benefits for caregiver's perceived relationship quality, their coping skills or sense of empowerment (Martin‐Cook et al., 2005). An RCT assessing the outcomes of aggression prevention training, encompassing dementia education, pain management, communication and behavioural activation did not find any benefits for relationship quality nor reduction in aggressive behaviours (Kunik et al., 2020). Jütten et al. (2018) trialled the use of a virtual reality dementia simulator as part of caregiver skills training, aiming to enhance caregivers' empathy for their loved one. However, whilst caregivers found the intervention informative and useful for developing caregiving strategies, no significant outcomes were found for couple's relationship quality.

Assistive technology is posited as an alternative means for supporting persons with dementia to maintain independence, reducing caregiver burden and delaying the need for residential care (Sriram et al., 2019). Whilst a systematic review by Sriram et al. (2019) identified how the use of assistive technology may strengthen relationships, they argue that the studies located were of variable quality and many lacked robustness. Since then, a recent UK RCT has been completed, involving a variety of health and social care professionals in the delivery of a full package of assistive technology and telecare for community‐dwelling persons with dementia (Gathercole et al., 2021). However, the study did not identify any outcomes for the couple's relationship and found no benefits for the person's quality of life, caregiver burden or mental health, and no delay in the requirement for transition to residential care (Gathercole et al., 2021).

#### 3. Connecting and strengthening the couple's relationship through sharing feelings and memories

Five of the studies located in the scoping review found positive outcomes for the couple's relationship from engaging in counselling therapy, enabling them to share feelings following dementia diagnosis and improve couple communication (Epstein et al., 2007; Larochette et al., 2019; Orsulic‐Jeras et al., 2019; Sørensen et al., 2008; Whitlatch et al., 2017). Couples' counselling in dementia care is aimed at couples where the ill spouse is in the early stages of dementia and able to actively engage with talking therapies (Epstein et al., 2007). The delivery of such psychotherapies requires specific counselling training and was provided by qualified counsellors and psychologists in each of the studies.

Reminiscence activities have already been demonstrated to be beneficial for the mental wellbeing of persons with dementia and to offer a small improvement in their cognitive functioning (Elfrink et al., 2018). A systematic review located 14 studies that used life story books, movie making and digital life story applications to support reminiscence work (Elfrink et al., 2018). The review concluded that many participants reported benefits for the couple's relationship and communication (Elfrink et al., 2018).

This scoping review identified studies utilising a dyadic approach to reminiscence, conducting activities with the person with dementia and their caregiver together. However, the outcomes of these studies were mixed.

A US study by Zarit et al. (2004) evaluated a memory club project for people with early‐stage dementia and their family caregivers, which provided dyads with the opportunity to meet and receive information and support for coping jointly. A survey conducted at the end of the intervention found the dyads enjoyed spending time together and it gave them the opportunity to share their feelings about the future and make plans together. Whilst not directly measured, the authors concluded that the intervention may have positive benefits in strengthening the dyadic relationship (Zarit et al., 2004). However, in contrast, an RCT of a 12‐week reminiscence group, ‘REMCARE’, found no support for the effectiveness of joint reminiscence groups and caregivers experienced a significant increase in stress and anxiety following participation (Charlesworth et al., 2016; Woods et al., 2012).

This scoping review identified a specific reminiscence intervention—life story books—which has consistently shown positive outcomes for the couple's relationship. Four of the studies found in the scoping review cover an intervention called the ‘Couple's Life Story Approach’, developed in the US. The 5‐week programme involves sessions using family photos to reflect on the couple's life together, improving communication and culminating in the production of a life story book (Ha et al., 2018; Ingersoll‐Dayton et al., 2013; Kwak et al., 2018). Caregivers reported that the intervention increased meaningful engagement with their partners, and social workers observed an increase in intimacy, as couples shared mutual memories (Ingersoll‐Dayton et al., 2013). However, some caregivers found the reminiscence process also acted as a painful reminder of what they had lost in their relationship since the onset of dementia (Ingersoll‐Dayton et al., 2013; Scherrer et al., 2013). Additionally, some caregivers found it difficult to compile items for the book and felt the intervention was best suited to early‐stage dementia (Ingersoll‐Dayton et al., 2013). The study used analysis of case studies, however, no assessment of the couple's relationship, before or after was completed, so it is not possible to measure the difference made or make comparisons with other interventions.

A web‐based tool has also been developed for couples to complete life story work (Sweeney et al., 2020). Participants found the tool useful in helping them to structure reminiscence activities with their partner and this strengthened their relationship. However, they also reported finding it time‐consuming to complete, and that the process of reminiscence elicited feelings of grief and loss (Sweeney et al., 2020). An app, called ‘InspireD’, has also been developed to offer personalised reminiscence for dementia caregiving dyads in the home (Laird et al., 2018; Ryan et al., 2020). Users of the app, reported it offered a useful aid to communication, sharing memories and increased feelings of closeness in the relationship (Ryan et al., 2020). The study found that app users with dementia benefitted from a statistically significant increase in mutuality, relationship quality and subjective wellbeing, however, there was no significant impact on caregivers (Laird et al., 2018).

## 4 DISCUSSION

This scoping review has highlighted a range of interventions that may support the couple living with dementia to maintain or enhance the quality of their relationship. The included papers highlight how leisure, arts, music and physical activities may be adapted to enable couples to engage in shared and meaningful activities, reduce social isolation and offer a respite from the caregiving role. The most frequently studied activity type was the use of music and singing activities. Those that demonstrated positive outcomes for the couple's relationship from engagement with music and singing activities were those that were (a) able to provide not only enjoyable but also equitable and meaningful interaction for the couple; (b) interventions designed to support continued participation within the home environment or (c) home‐based music therapy with ongoing support to the couple from a music therapist. Where music and singing were incorporated into the couples' day‐to‐day lives, additional benefits included a calming effect for the person with dementia, reduction in behavioural disturbances (Raglio et al., 2016) and calmer and easier completion of daily care (Melhuish et al., 2019).

Further research is required to ascertain the variables which differentiate why some interventions facilitating shared activity have been beneficial for the couple's relationship (e.g. Carbonneau et al., 2011) and others have been beneficial purely for caregiver wellbeing, without consequent impact on the couple's relationship (e.g. Leung et al., 2017).

Maintaining couple communication has been identified as vital to retaining relationship quality following dementia diagnosis. However, couples may need support to speak openly about the impact dementia has had on their role and identity (Epstein et al., 2007). Counselling programmes may offer couples an opportunity to communicate their feelings, experiences and concerns with their partner, opening up couple communication and enhancing relationship quality (Auclair et al., 2009; Epstein et al., 2007; Larochette et al., 2019; Orsulic‐Jeras et al., 2019; Sørensen et al., 2008; Whitlatch et al., 2017).

Reminiscence therapy has gradually become more prominent since the 1970s and is now one of the most popular non‐pharmacological interventions, designed to stimulate memory function for persons with dementia (Asano et al., 2021). However, the outcomes of reminiscence for the couple's relationship have been mixed. The most successful in terms of outcomes for the couple's relationship, is the ‘couple's life story approach’ in which couples compile a life story book together to reminisce. However, whilst reminiscence can increase meaningful and intimate engagement between partners, it can also become a painful reminder for caregivers of what they have lost as a result of the progression of their partner's dementia (Ingersoll‐Dayton et al., 2013; Scherrer et al., 2013).

There are a wide selection of existing community therapy interventions available for dementia caregivers that focus on delivering skills training and/or psychoeducation and reducing perceived caregiver burden. However, very few of these consider the impact of the intervention on relationship quality (Bielsten & Helström, 2017). This scoping review has highlighted a few programmes which offer caregiver psychoeducation and skills training and have identified positive outcomes for the couple's relationship. These studies found that through improving caregiver confidence in their knowledge and skills, there was a positive impact on caregiver emotional responses and interaction with their loved one, thereby improving relationship quality (Hepburn et al., 2003; Judge et al., 2009). However, further research is required to give more in‐depth explanation of how the programmes benefit the couple's relationship and identify if other existing caregiving skills programmes might also have secondary benefits for relationship quality.

Assistive technology may offer the opportunity to provide innovative support for people with dementia and their carers, within the current context of stretched health and social care resources (Sriram et al., 2019). A broad range of web or app‐based interventions are available to deliver arts, leisure and physical activity, self‐management, social connection, education and reminiscence activities to benefit the caregiver and care receiver and maintain or improve relationship quality. Positive outcomes for the couple may include improved interaction (Carbonneau et al., 2011), respite from caregiving activities and opportunity to connect with other caregivers (Lin et al., 2020), or providing a focus on time spent together (Tyack et al., 2017). However, several limitations have also been identified. Early provision of technology is important to ensure the caregiver has time to learn how to use the technology and facilitate it with their loved one; it is unlikely to be effective if delivered once the caregiver has already reached a crisis point (Yong et al., 2020). As dementia symptoms progress some users find it increasingly difficult to engage with the technology and this could add extra stress on the couple's relationship (Sriram et al., 2019). Additionally, not all caregivers feel comfortable communicating with other caregivers virtually and may favour face to face contact where possible (Lin et al., 2020). A recent UK RCT has also been unable to identify any outcomes for the couple's relationship from provision of assistive technology and telecare by health and social care professionals (Gathercole et al., 2021).

### 4.1 Reliance on verbal communication

Communication can become challenging as dementia progresses. As cognition is altered, the person with dementia is likely to increasingly withdraw from conversation with their partner and experience difficulty retaining spoken information, making conversation challenging and repetitive (Gillies, 2011). This impacts on the person's ability to participate in some types of intervention in the later stages of dementia (Letts et al., 2011). The majority of papers included in this scoping review were aimed at people with either mild or mild–moderate dementia. However, studies which stated their interventions were aimed at people inclusive of those with moderate to advanced dementia, utilised singing and music‐based interventions. This inclusiveness was discussed by Melhuish et al. (2019) who highlighted the value of the music therapy ‘Mindsong’, in its ability to enhance connection between partners, even once dementia had progressed into the more advanced stages. Youell et al. (2016) suggest that the use of nonverbal and intimate communication becomes increasingly important in the caregiving relationship as verbal communication functioning declines, suggesting supporting caregivers to utilise nonverbal communication skills may be an important area for future research.

### 4.2 Measuring outcomes for the couple's relationship

Whilst caregiving dyads recognise the importance of maintaining the relationship, a systematic review of psychosocial interventions found that this is generally not factored into the planning and design of dementia care interventions, and in particular the outcome measures selected (Rausch et al., 2017). It is notable that some of the interventions identified in this scoping review, such as the adaptation of leisure activities to enable persons with dementia to participate (Carbonneau et al., 2011) are very similar in approach to other existing programmes within community healthcare practice (e.g. Tailoring Activities for Persons with Dementia and Their Caregivers—TAP—Gitlin et al., 2016). Caregiver skills training programmes such as the ANSWERS programme (Judge et al., 2009) share some features in common with the COTiD‐UK programme (Wenborn et al., 2021) which did not examine outcomes for the couple's relationship. The key difference between studies included in this scoping review (which identify benefits for the couple's relationship) and other programmes aimed at persons with dementia and their informal caregivers, may not necessarily be the intervention type used, but rather the attention to, and measurement of, outcomes for the couple's relationship.

## 5 LIMITATIONS OF THE SCOPING REVIEW

This scoping review has been thorough in its search strategy, utilising a range of relevant databases, a wide range of search terms and a secondary examination of reference lists of studies has been completed. However, it is limited in its comprehensiveness by the exclusion of grey literature and studies published in languages other than English. This may have limited the transferability of the study to other cultures or excluded intervention methods trialled elsewhere.

There has not been a formal assessment of the methodological quality of the papers included (Joanna Briggs Institute, 2015). Therefore, whilst this scoping review offers an informative starting point for further research into potential community healthcare interventions, recommendations cannot be made for specific interventions.

## 6 RECOMMENDATIONS FOR FUTURE RESEARCH

This scoping review has highlighted a range of different intervention types which may have beneficial outcomes for relationship quality amongst couples experiencing dementia. Some of these interventions are designed to be delivered by facilitators outside of healthcare provision and further research is required to explore if it is possible or desirable to adapt these interventions for use by community healthcare practitioners.

Assessment and measurement of outcomes for the couple's relationship are currently both heterogenous and inconsistent, further studies are required to explore how couple's relationship outcomes can most effectively be captured, taking into account relationship quality pre‐ and post‐intervention, differentiation of caregiver types and client‐centred goals and outcomes. Future research could consider more in‐depth analysis of the methodology of these studies, how interventions are delivered and the variables involved in the generation of positive outcomes for the couple's relationship, where similar programmes have been less successful. An important area for future research also includes the study of existing, successful community programmes for couples experiencing dementia, which may also have currently unexplored, secondary benefits for the couple's relationship quality.

This scoping review has also highlighted the need for longitudinal studies to examine if the benefits of interventions aimed at those with mild–moderate dementia are sustained as dementia progresses into the moderate–severe stages. Research is also required to identify interventions less reliant on continuing verbal communication skills for the participation to continue as dementia progresses and how caregivers can utilise non‐verbal communication to improve couple interaction and closeness in the latter stages of dementia progression.

## 7 CONCLUSION

In conclusion, the scoping review has elucidated a variety of interventions that support the couple's relationship for people living with dementia, originating from a broad range of disciplines. The review has highlighted three key ways in which interventions may assist to maintain or enhance the quality of the couple's relationship. Firstly, through the adaptation of leisure, physical and arts activities to facilitate meaningful shared activity for the couple, or the use of music and singing activities both in community groups and at home to offer a calming effect on the person with dementia, and to provide more equitable and meaningful interaction. There is also increasing interest in the potential use of technology to support the identification of and participation in meaningful shared activities for the couple.

Secondly, the scoping review has highlighted the potential beneficial impact on the couple's relationship, of supporting couples to communicate openly with each other about the impact of dementia on both themselves and their relationship. Whilst generally delivered by trained counsellors, there may be skills and techniques which could be utilised by healthcare practitioners to support couples. The reminiscence intervention, the ‘Couple's Life Story Approach’, has shown positive outcomes for couples through supporting them to engage in meaningful and intimate communication and reminiscence together, and has potential for adaptation by various health and social care practitioners. However, it is important that practitioners consider that participation in reminiscence can also generate a painful grief response for some couples.

Thirdly, some caregiver education and skills training programmes have identified not only a reduction in caregivers' perceived sense of burden, but a consequent improvement in their interaction with their partner which has enhanced relationship quality. This suggests that other existing programmes outside of this scoping review which aim to reduce caregiver burden, may also have outcomes for couple interaction and relationship quality, which are as yet unexplored and could be enhanced further.

The scoping review also highlights the need for further studies to explore if it is possible or desirable to adapt some of these interventions for use by healthcare practitioners in the community. There is a need for further research to identify interventions that are appropriate to support the couple's relationship in the moderate‐advanced stages of dementia. Additionally, researchers may want to consider how outcomes for the couple's relationship may be most effectively measured and captured given the diversity and inconsistency in outcomes measures currently in use.

## AUTHOR CONTRIBUTIONS

All authors (Colloby, Whiting & Warren) have made substantial contributions to conception and design, acquisition of data, analysis and interpretation of data, been involved in drafting the manuscript or revising it critically for important intellectual content, given final approval of the version to be published. Each author has participated sufficiently in the work to take public responsibility for appropriate portions of the content and also agreed to be accountable for all aspects of the work in ensuring that questions related to the accuracy or integrity of any part of the work are appropriately investigated and resolved.

## CONFLICT OF INTEREST

No conflicts of interest.

## Supporting information


Appendix S1

## Data Availability

Data sharing not applicable to this article as no datasets were generated or analysed during the current study.
