# Supplementary material for: Supporting the couple relationship following dementia diagnosis: A scoping review
Source: Health Soc Care Community. 2022 Sep 20;30(6):e3643–55. doi: 10.1111/hsc.14006 (PMC10087355; doi:10.1111/hsc.14006)
Supplement: Supplementary file 1 — Appendix S1 [file HSC-30-e3643-s001.docx]

**Appendix 1 - Inclusion and exclusion criteria**



| **Selection criteria** | **Inclusion** | **Exclusion** |
| --- | --- | --- |
| **Population** | Participants include members of couples (married or cohabiting, heterosexual or homosexual), where one partner has a diagnosis of dementia. | Studies that focused on persons with dementia living in residential care |
|  |  | Studies that focussed on relationship with paid carers |
|  |  | Studies including conditions other than dementia |
|  |  | Studies that focussed solely on outcomes for person with dementia OR caregiver |
| **Intervention or approach** | Interventions delivered at home, hospital or community-settings |  |
|  | Interventions aimed at improving the couple relationship or citing outcomes related to improvement or sustenance of couple relationship. |  |
| **Study design** | All types of methodology, including research synthesis, pilot studies | Grey literature |
|  | Studies from all fields outside of occupational therapy | Studies that focused on comparison of dementia with other conditions |
|  | Studies undertaken in any country | Studies focussed on couple experience and did not explore interventions |
| **Date and language restrictions** |  | Studies written in languages other than English (due to time restrictions and resources available for translation). |
|  |  | Studies published prior to year 2000 (due to volume of records retrieved for screening, and limited time and resource capacity for screening of records). |

**Appendix 2 - Data charting table**

| **Author(s)** | **Study aims/purpose** | **Population/ sample size** | **Methodology/ research method** | **Intervention type, duration** | **Intended for intervention by [professional]** | **Outcome tool and outcomes** for relationship | **Other key findings** |
| --- | --- | --- | --- | --- | --- | --- | --- |
| **Arts-based interventions** |  |  |  |  |  |  |  |
| Hunt, Truran & Reynolds (2018) | Explore carer experience from existing participation in art making activities | 6 female family caregivers for persons with dementia (4 x spousal, 2 x caring for mother) | Semi-structured interviews with Interpretative Phenomenological Analysis (IPA) | Regular engagement in art and craft activities at community groups and privately at home. | Not stated | IPA analysis of themes and meaning  Participants reported greater resilience in caring relationship – and greater social connection with care recipient | Arts activities offered respite, and improved connection with community outside of caregiving. |
| Tyack, et al., (2017) | Explore if arts based interventions can be facilitated via touchscreen tablet | 12 dyads – persons with dementia and informal caregivers | Mixed methods – interviews, thematic analysis, wellbeing measure. | Participants trialled use of tablet for 2 weeks following introduction to study. Asked to use tablet at least 5 times. | On tablet | QOL-AD, interview transcript analysis  Some participants identified a positive benefit for the relationship – enabling deeper conversation, focussed time together. | Significant improvement in wellbeing. |
| **Assistive technology** |  |  |  |  |  |  |  |
| Bielsten, et al., (2020) | Explore experience of couples engaging with the DemPower app | 6 Swedish couples in long term relationship living at home, where one partner has dementia diagnosis | Semi-structured interviews, thematic analysis | App on tablet – for couples to engage with together at home and develop self-management techniques | Delivered by app | Semi-structured interviews – positive impact on couple relationship, togetherness and communication.  Thematic analysis. | Couple’s reported using app initially felt uncomfortable, but then helped their relationship, enabling them to talk more openly, try new activities and feel closer |
| Sriram, Jenkinson, & Peters (2019) | Explore the outcomes of assistive technology use for carers of persons with dementia living in the community | 56 publications – includes paid and family caregivers | Systematic review and thematic analysis. Includes mix of qualitative, quantitative and mixed methods studies | 84 types of assistive technology including GPS trackers, timers/reminders, reminiscence tools, robotics, sensors, safety alarms, communications aids | Not stated | Thematic analysis- Using assistive technology to enhance leisure, social interaction, orientation, safety and provide memory support appeared to actively strengthen the relationship between person with dementia and carers | When the technology failed or person with demented no longer able to utilise it, this impacted the relationship with carers. |
| **Caregiver training/ psychoeducation** |  |  |  |  |  |  |  |
| Au, et al., (2019) | Evaluate impact of telephone-based psycho-education for dementia family caregivers | 96 family caregivers for persons with dementia | Double-blinded Randomized Controlled Trial + qualitative feedback | 4 x weekly telephone based psychoeducation sessions + 8 x biweekly sessions of behavioural activation.  Control group – 4 x psycho-education sessions + 8 sessions of general monitoring | Social worker, paraprofessional coaches | Questionnaires, follow up call, 7 item Relationship Assessment Scale, Zarit Burden Interview, Self-efficacy for controlling upsetting thoughts (SE-CU) scale.  Programme enhanced relationship satisfaction amongst caregivers. | Programme reduced perceived burden and symptoms of depression.  Telephone delivery suitable for psychoeducation and psychotherapeutic intervention |
| Hepburn, et al., (2003) | Testing the feasibility of translating this psychoeducational programme for caregivers into different settings and locations. | 140 family caregivers | Three site field trial (quasi-experimental), pre intervention baseline assessment of caregiver burden, wellbeing and mental health. Repeated again at 3-4 months after beginning of intervention. Evaluation forms completed by participants at each session. | 12 hour dementia centre-based course for caregivers – focussed on caregiver role, skills, attitudes and self-care. | Variety of professional background | Revised memory + behaviour checklist, Caregiver Burden Scale, Centre for Epidemiologic Studies – Depression Scale, Mastery Scale.  Improvement in caregiver emotional response to caregiving – not explicitly relationship | Improvement in caregiver confidence, response to care receiver behaviours, reduction in perceived burden.  Programme flexible enough to be delivered by range of professionals. |
| Judge et al. (2012) | Assess efficacy of intervention ‘ANSWERS’ – Acquiring New Skills While Enhancing Remaining Strengths | 128 dyads (60% spousal caregivers) | Randomized controlled trial | ANSWERS programme - 6 x skills training sessions with dyads, focused on dementia, memory loss, communication, staying active, emotions and behaviours | Masters-level intervention specialist | Measure of dyadic relationship strain (Bass, Tausig & Noekler, 1989). Measures of emotional health strain, caregiver mastery, depression, anxiety, self-esteem and quality of life.  Reduced carer strain, dyadic relationship strain | Higher sense of caregiving mastery, improved emotional health of caregivers. |
| Jütten, Mark & Sitskoorn (2018) | Evaluate impact on caregiver wellbeing, and relationship quality of a virtual reality programme for informal caregivers | 201 informal caregivers | Quasi-experimental longitudinal study, semi-structured interviews, questionnaires | D’mentia - Virtual reality dementia simulator designed to enable participant to experience what it is like to have dementia. 1 to 1 support with trainer. Group discussion. | Not specified | Interpersonal Reactivity Index (IRI), Caregiver Reaction Assessment, Hospital Anxiety & Depression Scale, Relationship Quality Index, Quality of Relationship Scale, Short Sense of Competence Questionnaire.  No significant impact identified in relationship quality | Caregivers found intervention informative and useful for caregiving strategies. Outcomes tools did not identify any impact on perceived burden, anxiety, depression, sense of competence or empathy. |
| Kunik, et al., (2020) | To evaluate aggression prevention training programme for persons with dementia and family caregivers | 228 caregiver – care receiver dyads | Single blind randomized controlled trial | Skills-based aggression prevention training for person with dementia and caregiver delivered over 3 months in home + follow up telephone calls | Not specified | CMAI aggression scale, Geriatric Anxiety Index (short form), Geriatric Depression Scale, Zarit Burden Scale, 15 item Mutuality Scale, Revised Memory and Behavior Checklist  No change or benefits found for relationship quality, decrease in those receiving usual care | No benefits or change found in all outcomes assessed. |
| Martin-Cook et al., (2005) | Examine the impact of caregiver skills programme aimed at improving relationship quality between family caregivers and persons with dementia. | 47 caregiving dyads – persons with mild-moderate dementia. | Randomized controlled trial, half of sample randomized in an intervention group, other half remaining on waiting list. | 4 weekly caregiver skills training sessions, aimed at aligning caregiver expectations and their loved one’s actual functioning abilities. Offering training on using cues and prompts to facilitate performance, breaking down tasks, and communication skills.  Non-dyadic | Not specified | Relationship attribution measure carried out with caregivers. Other measures: Mini-mental status examination (MMSE), Geriatric Depression Scale, Independent Living Scale, Alzheimer’s Disease Cooperative Study – Activities of Daily Living Inventory – MCI version, Texas Functional Living Scale, Neuropsychiatric Inventory, Finding meaning through caregiving scale.  Measured at baseline, repeated at week 7 + week 17.  No significant improvement in caregivers’ perception of the relationship | Any improvement in congruence between caregiver expectations and the care receiver’s functioning did not persist over course of study.  No significant improvement in caregiver coping skills or empowerment. |
| Zarit, et al., (2004) | Project evaluation of group intervention for persons with dementia and caregivers | 23 dyads – persons with early stage dementia and care partners | Project evaluation – participant feedback survey | 10 group sessions for dyads to meet, receive information and support for coping. | Social worker, neuropsychologist | Feedback results – supported dyads in addressing issues affecting them and reducing conflict. Provided opportunity to share feelings about the future and plan jointly. | Authors concluded this may strengthen dyad although outcome not specifically measured. |
| **Cognitive Rehabilitation Therapy** |  |  |  |  |  |  |  |
| Leung, Orgeta, & Orrell (2017) | Examine impact on carer wellbeing following cognitive based interventions for care receiver. | Meta analysis included 7 studies (total of 803 dementia caregiving dyads) | Meta analysis of randomized controlled trials | Interventions involving cognitive rehabilitation, stimulation or training | Not specified | Outcomes measurements included those of carer well-being, quality of life, mood, physical and mental health.  Caregiving relationship and perceived caregiver burden.  2 studies included assessed outcomes for relationship – no significant improvement found. | Significant improvement in caregiver quality of life and reduction in caregiver anxiety. |
| **Counselling** |  |  |  |  |  |  |  |
| Auclair, Epstein & Mittelman (2009) | Evaluate the value of counselling for dementia caregiving couples and qualitative outcomes. Build on the earlier pilot study. | 30 couples with one partner experiencing early stage dementia. | Randomized wait-list control design – immediate treatment group vs. group offered same intervention following 4 month evaluation. | 6 counselling sessions using Gestalt Therapy, Transactional Analysis. Aimed at supporting couple relationship and sense of self. Couple participating as equals in therapy. | Counsellors | Evaluations at baseline, 2 and 4 months – written questionnaires conducted separately with each partner. Dyadic adjustment scale, Goal attainment scale.  Relationship strengthened by couples developing greater acceptance and non-judgmental attitudes. | 6 counselling sessions appears sufficient to move couple forwards |
| Epstein, Auclair & Mittelman (2007) | Evaluate the value of counselling for dementia caregiving couples | 10 couples – partner with early stage alzheimer’s disease (able to understand their situation and communicate effectively). | Pilot study – randomized wait list control | 6 dyadic counselling sessions over period of 2 months utilising elements of CBT and psychodynamic therapies. | Counsellor | Dyadic adjustment scale (marriage quality), Goal Attainment Scale  Counselling enhanced feelings of togetherness, empowering, improved positive interaction and optimism. |  |
| Larochette, et al., (2019) | To assess outcome of support program | 23 spousal caregivers for people living with young onset dementia (under 65 years) | Semi-structured interviews, questionnaires and thematic analysis | 7 x 90 minute sessions of acceptance and commitment therapy with caregivers in their homes | Psychologist trained in acceptance and commitment therapy | Thematic analysis – caregivers found greater meaning in their relationship and acceptance of their partner’s diagnosis. | Helped caregivers to express, manage and reduce impact of difficult thoughts and emotions, discover new caregiving strategies, and improved communication. |
| Sorensen, Waldorff & Waldemar (2008) | Analysis of participant experiences and outcome following participation in intervention | 10 dementia caregiving couples | Semi-structured interviews pre/post intervention | Danish Alzheimer Intervention Study (DAISY) – home delivered tailored counselling support over 6 months + support group aimed at caregiver and person with dementia | Not specified | Thematic analysis - Caregivers reported improved mutual communication, and more easily able to approach daily challenges and interaction | Participants found the programme rewarding, stimulating, and enjoyed spending time with others in support group |
| Whitlach, et al., (2017) | Feasibility and acceptability trial of dyadic care planning intervention. | 128 dyads experiencing early stage dementia | Randomized controlled trial. | SHARE program – 6 sessions of counselling/psychoeducational intervention in participants homes - for family caregivers and receivers – aimed at enhancing communication patterns, managing emotional impact of diagnosis and care planning. | Counsellors/social workers | Results of trial suggest improvement in dyadic relationship functioning. | Couples able to complete effective forward care planning together. |
| **Multi-modal programmes** |  |  |  |  |  |  |  |
| Carbonneau, Caron & Desrosiers (2011) | To evaluate impact of an adapted leisure education program for dementia caregivers | 49 dyads – persons with Alzheimer’s Disease and family caregivers (30% spousal) | Mixed methods – pre/post test assessment, open ended interviews | Leisure education programme – 4-6 sessions + follow up telephone calls. Education and shared leisure activities. | Not specified | General well-being schedule, Relationships in Elder Care scale, interview transcript analysis.  Statistically significant improvement in relationship quality. Participants reported experiencing pleasurable moments with care receiver, improved interaction. | Caregivers reported improved quality of life for care receivers. No significant change in caregiver wellbeing, but significant improvement in caregiver self-efficacy. |
| **Music and singing** |  |  |  |  |  |  |  |
| Allan (2018) | Explore effectiveness of intervention for meeting needs of couple for community, relationships | 3 couples including person with dementia | Mixed methods – pre/post questionnaire, thematic analysis of clinical notes, volunteer feedback, evaluation forms | 10 themed weekly group sessions for couples, singing, dancing, percussion, sharing photographs | Music therapist | Feedback questionnaires, rating scale (1-10) – Participants experienced no improvement in wellbeing, emotional connectedness, decline in coping and enjoyment | Participants valued support, reminiscence and sharing with other couples. Participants found it difficult to measure other outcomes. |
| Baker, Grocke & Pachana (2012) | Examine relationship outcomes for caregivers following program | 5 couples living at home (including partner with dementia) | Mixed methods – participant diaries, assessment questionnaire and semi-structured interviews | Home based 6 week active music intervention – singing, gentle movement, listening to music for 20 mins – followed by reminiscence | Music therapist | Mutual Communal Behaviors Scale, Positive aspects of caregiving questionnaire, Geriatric Depression Scale (short form), Geriatric Anxiety Inventory – participants reported some improvement in closeness in relationship and reciprocity, enjoyed time spent together. | Participant relationship quality already high and depression and anxiety low, study found ceiling effect. Participants enjoyed the program and relaxing together. Reported being able to use in home environment beneficial. |
| Baker et al., (2019) | Protocol – study into caregiver-delivered music intervention | Dyads (cohabiting family member or friends) for people with dementia | Protocol for Randomised Controlled Trial | Homeside – 2 hour home-based musical intervention training session for using music to help calm person with dementia, following by further 2 training sessions at 3 + 6 weeks. Fortnightly telephone support to caregivers | Music therapist | Person with dementia - NPI-Q (severity of behaviours), MMSE, Qol-AD, MADRS (depression scale).  Caregiver – PHQ-9, Caregiver resilience scale, Short sense of competence scale, Quality of Life – 6D, 14 item – Quality of Caregiver-Patient Relationship scale | Results not yet published. |
| Clark, Tamplin & Baker (2018) | Explore participants’ experience of therapeutic group singing intervention | 9 persons with dementia and 9 family caregivers | Semi-structured dyadic interviews + thematic analysis | 20 x weekly therapeutic group singing sessions attended by both members of dyad, provision of music based resources for home use | Music Therapist | Analysis of interview transcript – promoted feelings of togetherness and evoked meaningful memories.  Dyads enjoyed participating together. | Made singing more accessible, boosted participant mood. |
| Clark et al., (2020) | Examine acceptability and outcomes of dyadic therapeutic song writing intervention | 10 dyads (mostly spousal) including member with dementia | Pre-post test feasibility study | 6 week, 1 hour therapeutic songwriting group for dyads | Music Therapist | Quality of the Caregiver – Patient Relationship (QCPR Primary), Cornell Scale for Depression in Dementia (CSDD), PHQ-9, AQol – 8D, Zarit Burden Interview, Quality of Life – Alzheimer’s Dementia scale  Relationship quality high at baseline and remained so, qualitative data indicated the intervention supported the relationship rather than changing it’s quality. | Significant increase in quality of life for both members of dyad and reduction in depression in participants with dementia.  Design and delivery were acceptable to participants. |
| Dassa, Rosenbach & Gilboa (2020) | Assess impact of intervention model on sustainability of incorporation of music in daily life. Identify recommendations for implementation of model. | Pilot with 2 couples | Case study | 12, weekly home-based music therapy sessions with couple + fortnightly counselling sessions with caregiver | Music therapist | Participants reported enjoyment of activities together, laughter, and moments of equality in relationship. | Provided respite from daily frustrations |
| Lee, O’Neill & Moss (2020) | To explore how group singing intervention impacts wellbeing | 3 persons with early stage dementia and 4 family caregivers. | Semi-structured interviews – Interpretative Phenomenological Analysis | 6 x weekly 1 hour group singing sessions at community arts centre | Music therapist | Themes drawn from Interpretative Phenomenological Analysis – participants found it offered an opportunity to spend meaningful time together, not just in a caring/ cared for role. | Group offered a sense of belonging, feelings of rejuvenation and participants enjoyed collaboration with others. |
| Melhuish, Grady & Holland (2019) | Evaluate effectiveness of home music therapy intervention for couples experiencing dementia | 7 people with advanced dementia and family carers | Mixed methods – scaling tool, carer’s checklist and feedback questionnaire+ case study | 12 music therapy sessions delivered at home | Music Therapist | Bespoke scaling tool for completion by therapist – engagement, well-being and relationships. Mental Health Foundation Carer’s checklist. Carer feedback questionnaire.  Carers felt more connected with their partner and greater feeling of intimacy.  Improvement in relationship scores | 73% showed some improvement in well-being.  Helped carers extend support networks.  Music therapy usually held in residential care can also work well in the home. |
| Osman, Tischler & Schneider (2016) | Examine the impact of group singing activity ‘Singing for the Brain’ for people with dementia and their carers. | 10 pairs of participant with one member with dementia – 7 husband-wife, 3 mother-daughter. | Semi-structured dyadic interviews covering participants experience of attending the group, participation, impact on communication and the relationship. | 2 month period of attending group singing activities and peer support. | Musician | Thematic analysis.  Participants reported reduction in feelings of social isolation, mutually enjoyable, offered meaningful shared interaction, positive impact on caregiver and receiver wellbeing. | Participants reported finding it achievable and enjoyable, even once the person with dementia was no longer able to participate in many of their usual ADLs. |
| Raglio et al., (2016) | Investigate effectiveness of Active Music Therapy in reducing behavioural disturbances in people with dementia and burden on family caregivers | 4 couples experiencing dementia | Completion of psychological assessment, pre intervention, post treatment and at 1 month following end of intervention. | 12 x 40 min sessions of active music therapy, twice weekly, aimed at promoting communication and couple relationship | Music Therapist | Burden interview, Hamilton Anxiety Rating Scale, Beck Depression Inventory, Geriatric Depression Scale.  Authors conclude – may support relationship, but not measured directly in outcomes | Caregivers reported intervention supported communication, reduced anxiety, depression, burden. Reduction in behavioural disturbances and depression in person with dementia. |
| Skingley et al., (2020) | Project evaluation – community music group | 16 family caregivers for persons with dementia | Focus group, thematic analysis | Community-based creative singing group for people with dementia and their carers | Not specified | Thematic analysis of focus group – Caregivers felt more in touch with their loved one and enjoyed activity together. | Caregivers reported improved relaxation and found sessions an escape from caregiving duties. Caregivers reported calming effect for participants with dementia. Caregivers continued using singing at home with partner during activities of daily living. |
| **Physical activity-based interventions** |  |  |  |  |  |  |  |
| Casey et al., (2020) | Examine feedback from group movement programme for people with dementia and their carers | 9 dyads – persons with mild-moderate dementia and their family or paid caregivers | Participant feedback, daily logs – qualitative coding system | Programme integrating physical movement sequence, cognitive stimulation, social engagement, caregiver training. – community class – 12 weeks | Not specified | Feedback analysis – Caregivers reported improvement in relationship quality and feelings of closeness. | Caregivers reported improvement in physical and emotional functioning, social connection and reduced stress.  Randomized controlled trial in progress. |
| Lin et al., (2020) | Explore impact of social exergames on caregiver physical activity and social connectedness | 18 dementia family caregivers | Mixed methods – semi-structured interviews, app usage logs, grounded theory, inductive thematic analysis. | Go & Grow - 6 week evaluation of smart phone app programme of fitness challenges for caregivers. Social platform to share experience with other caregivers. | App-based | Thematic analysis – caregivers reported increased empathy for loved one, improved interaction and some reported increased patience. | Some caregivers reluctant to share challenges faced with others they had not met in real life.  Caregivers reported reduced stress and increased physical activity. |
| **Reminiscence-based interventions** |  |  |  |  |  |  |  |
| Charlesworth et al., (2016) | To evaluate programme of peer support and reminiscence therapy | 291 pairs | Factorial pragmatic randomised trial | 12 x weekly 2 hour group sessions – covering drama, singing, and peer support. Followed by 7 x monthly sessions, including caregiver sessions on communication skills and activities for home. | Not specified | SF-12, QOL-AD, EQ-5D, HADS, Emotional Loneliness Scale, Caregiver Distress Scale, QCPR (Quality of Caregiver-Patient Relationship), Positive Aspects of Caregiving, COPE Index, Personal Growth Index, DemQOL. Alzheimer’s Disease Cooperative Study – Activities of Daily Living Inventory (ADCS-ADL).  At 12 months there was improvement in carers perception of quality of relationship. | No significant benefits found in outcomes measurement for caregivers quality of life. |
| Elfrink et al., (2018) | Review how life story books are used and type of studies conducted to evaluate their use. | 14 studies met inclusion criteria – included a total of 243 persons with dementia | Systematic Review | Creating life story books, movie making, physical books and digital applications. | Not stated | Large variety of outcome measures used.  Participants found reminiscence beneficial to relationship – providing affirmation and improved interaction. | Participants found creating life story books enjoyable. |
| Ha et al., (2018) | Evaluate the Korean adaptation of the Couple’s Life Story Approach | 37 couples experiencing dementia | Pre + post test psychosocial outcomes measures | Couple’s Life Story Approach – using photos to reflect on marriage history and improve communication skills. | Social worker | Mutuality scale, Geriatric Depression  Scale, 12 item Caregiver Burden Scale  Small increase in mutuality in younger couples but reduction in older couples. | Some couples were more interested in improving cognitive function, rather than relationship quality. |
| Ingersoll-Dayton et al., (2013) | Examine feasibility and outcomes for participant of a couples life story approach | 20 couples experiencing dementia | Feedback questionnaire | Couple’s Life Story Approach – 5 x 1 hour (usually weekly) with both partners. Sessions covering communication skills and reviewing stages of life together, creating life story book. | Social worker | Questionnaire – Caregivers reported intervention enabled meaningful engagement as a couple, social workers observed couples became more intimate during process. | Some caregivers had difficulty compiling items for book. Some caregivers found reminiscence painful reminder of aspects of relationship lost.  Some caregivers found more effective in early stage dementia. |
| Ingersoll-Dayton et al., (2016) | Explore clinical themes arising from this intervention with couples in US + Japan | 20 couples in US experiencing dementia and 9 in Japan | Case study | Couples Life Story Approach – 5 week intervention, home-based, creating life story book and provision of communication tips | Social worker | Case study analysis – couples reported that it gave them opportunity to recognise each other’s strengths and the positive aspects of their relationship, and the richness of their shared lives. | Couples were supported in reminiscing about difficult as well as positive life events. Provided opportunity for couples to relate to each other in new ways. |
| Kwak, Han & Ha (2018) | To understand how Korean couples experience engaging with the Couples Life Story Approach | 56 couples experiencing mild alzheimer’s disease. | Questionnaire, thematic analysis | Couples Life Story Approach - 5 weekly sessions reviewing periods of their life, creating a life story book and developing communication skills | Not stated | Analysis of questionnaire responses  Participants reported feeling closer bond, valued opportunity to hear about their partners feelings and thoughts, and appreciate each other and their life together.  Increase in patience and understanding for their partner. | Majority of participant enjoyed reminiscence - some participant experienced revival of negative emotions such as guilt, bitterness and loss.  Using photos aided reminiscence. |
| Laird et al., (2018) | Measure impact of reminiscence app on closeness and quality of relationship and well-being. | 29 dyads experiencing early-moderate dementia. | Outcome measurement at baseline, midpoint and post intervention | 19 week reminiscence intervention, delivered via bespoke tablet app | Not specified | 15 item mutuality scale, QCPR (relationship quality scale), WHO-5  Statistically significant increase in relationship quality, mutuality and subjective well-being amongst persons with dementia.  Non significant improvement for carers | Reminiscence activities were able to be successfully delivered via an app |
| Ryan et al., (2020) | Explore impact of reminiscence app on people with dementia and their family carers | 15 persons with dementia and 17 family carers | Semi-structured interviews, thematic analysis | InspireD app for tablet, for home use, providing personalised reminiscence for persons with dementia to use with family carers | Not specified | Analysis of interview themes – participants reported feeling closer to their loved ones, helped them to share memories, be more open and improved communication. Opportunity to celebrate their shared lives. Caregivers reported increased patience and respect with their loved one. | Use of personalised reminiscence app can have positive impact on persons with dementia and carers both individually and in their relationship. |
| Scherrer, Ingersoll-Dayton & Spencer (2013) | Explore challenges in using couple’s life story approach and strategies to address these. | 20 older couples experiencing memory loss | Case study, narrative analysis | 5 week structured dyadic intervention, narrating a life story together – delivered in participant’s home | Social worker | Narrative analysis – improvement in couples’ communication. | Participants found it a positive experience overall but revisiting difficult life events for challenging for couples |
| Sweeney, Wolverson & Clarke (2020) | Examine impact of digital life story book making on couple wellbeing | 4 couples experiencing dementia | Semi-structured interviews and thematic analysis | 6 week programme creating digital life story book as a couple, via website. | Not specified | Analysis of interview transcripts – Some participants reported programme helped them to make sense of their feelings, and flourish and grow as a couple. | Participants also found it time-consuming to put together.  Some experiences were mixed with reminiscence also evoking feelings of loss. |
| Woods et al., (2012) | Evaluate effectiveness and cost-effectiveness of reminiscence groups for people with dementia and family caregivers. | 350 dyads, 71% spousal | Multicentre Randomised Controlled Trial | 12 x weekly reminiscence groups for caregiver and care receiver together. Followed by monthly maintenance sessions for 7 months. | Not specified | General Health Questionnaire, QOL-AD scale. QCPR (quality of relationship scale). Autobiographical memory interview. Rating Anxiety in Dementia (RAID). Hospital Anxiety and Depression Scale (HADS). Cornell Scale for Depression in Dementia.  Although there may have been some benefits for participant, participants had raised anxiety and stress. Trial not supportive of effectiveness of joint reminiscence groups. |  |
| **Reviews (mixed intervention types)** |  |  |  |  |  |  |  |
| Moon & Adams (2013) | Review impact of dyadic interventions for persons with early stage dementia and their caregivers | Included 12 studies (total 489 Dyads) | Critical Review | Dyadic interventions – cognitive stimulation, memory clubs, support groups, counselling, education programmes, skills training. | Not stated | Variety of outcome measures used.  Themes identified – Reviewed studies indicate dyadic interventions can enhance relationship quality, communication and mutual understanding. | Improvement in care receiver’s wellbeing. Increase in caregiver knowledge and coping skills. |
| Rausch, Caljouw & van der Ploeg (2017) | Explore dyadic psychosocial interventions available for persons with dementia and their informal caregivers | 6 studies included | Systematic Review | Psychosocial interventions delivered to person with dementia and caregiver together | Not specified | Studies used a variety of outcome measures. | Studies included had low statistical power, did not account for differences in relationship type (e.g. spousal/child-parent), most found positive qualitative results, but were unable to detect changes in quantitative outcomes measures |

**Appendix 3 - Outcome measures for relationship quality**

| **Measures of relationship quality** |
| --- |
| 7-item relationship assessment scale (Hendrick, 1988) |
| Dyadic Adjustment Scale (Spanier, 1976) |
| Interpersonal Reactivity Index (IRI) (Davis, 1980) |
| Measure of elder impairment, social support and caregiver strain (Bass, Tausig & Noelker, 1989) |
| Mutual Communal Behaviors Scale (Williamson & Schulz, 1990) |
| Mutuality Scale (Archbold & Stewart, 1986) |
| Quality of Caregiver-Patient Relationship Scale (QCPR) (Spuytte et al., 2002) |
| Quality of Relationship Scale (Bengtson, 2009) |
| Relationship Attribution Measure (Fincham & Bradbury, 1992) |
| Relationship Quality Index (Norton, 1983) |
| Relationships in Elder Care Scale (Lyonette & Yardley, 2003) |

**Appendix 4 – Participants’ stage of dementia and intervention type**

| **Stage of dementia** | **Arts-based** | **Assistive technology** | **Caregiver skills training** | **Cognitive rehabilitation** | **Counselling** | **Multi-modal** | **Music/singing** | **Physical activity** | **Reminiscence** | **Reviews** | **Total** |
| --- | --- | --- | --- | --- | --- | --- | --- | --- | --- | --- | --- |
| Early/mild |  |  | 1 |  | 4 |  | 1 |  | 4 | 1 | 11 |
| Mild-moderate |  |  | 3 | 1 |  |  | 1 | 1 | 6 | 1 | 13 |
| All stages |  |  |  |  |  | 1 | 3 |  |  |  | 4 |
| Not stated | 1 | 2 |  |  | 1 |  | 6 |  | 1 |  | 11 |
| Caregivers only | 1 |  | 3 |  |  |  |  | 1 |  |  | 5 |
